# An RS Motif within the Epstein-Barr Virus BLRF2 Tegument Protein Is Phosphorylated by SRPK2 and Is Important for Viral Replication

**DOI:** 10.1371/journal.pone.0053512

**Published:** 2013-01-09

**Authors:** Melissa Duarte, Lili Wang, Michael A. Calderwood, Guillaume Adelmant, Makoto Ohashi, Jennifer Roecklein-Canfield, Jarrod A. Marto, David E. Hill, Hongyu Deng, Eric Johannsen

**Affiliations:** 1 The Channing Laboratory, Department of Medicine, Brigham and Women’s Hospital, Harvard Medical School, Boston, Massachusetts, United States of America; 2 Center for Cancer Systems Biology (CCSB) and Department of Cancer Biology, Dana-Farber Cancer Institute, Boston, Massachusetts, United States of America; 3 Key Laboratory of Infection and Immunity, Institute of Biophysics, Chinese Academy of Sciences, Beijing, China; 4 Department of Genetics, Harvard Medical School, Boston, Massachusetts, United States of America; 5 The Blais Proteomics Center, Department of Cancer Biology, Dana-Farber Cancer Institute, Boston, Massachusetts, United States of America; 6 Department of Biological Chemistry and Molecular Pharmacology, Harvard Medical School, Boston, Massachusetts, United States of America; 7 Department of Medicine, University of Wisconsin, Madison, Wisconsin, United States of America; 8 Department of Chemistry and Physics, Simmons College, Boston, Massachusetts, United States of America; The University of North Carolina at Chapel Hill, United States of America

## Abstract

Epstein-Barr virus (EBV) is a gammaherpesvirus that causes infectious mononucleosis, B cell lymphomas, and nasopharyngeal carcinoma. Many of the genes required for EBV virion morphogenesis are found in all herpesviruses, but some are specific to gammaherpesviruses. One of these gamma-specific genes, BLRF2, encodes a tegument protein that has been shown to be essential for replication in other gammaherpesviruses. In this study, we identify BLRF2 interacting proteins using binary and co-complex protein assays. Serine/Arginine-rich Protein Kinase 2 (SRPK2) was identified by both assays and was further shown to phosphorylate an RS motif in the BLRF2 C-terminus. Mutation of this RS motif (S148A+S150A) abrogated the ability of BLRF2 to support replication of a murine gammaherpesvirus 68 genome lacking the BLRF2 homolog (ORF52). We conclude that the BLRF2 RS motif is phosphorylated by SRPK2 and is important for viral replication.

## Introduction

Epstein-Barr virus (EBV), the prototypical gammaherpesvirus, can cause infectious mononucleosis in healthy individuals, B-cell lymphoproliferative disease in immunosuppressed individuals, and rarely, B-cell lymphomas, Hodgkin lymphoma, and nasopharyngeal carcinoma in otherwise healthy people [1], [2]. Gammaherpesviruses, including EBV and Kaposi Sarcoma associated herpesvirus (KSHV), establish latent infections in cells and their associated malignancies are a by-product of the growth and survival signals triggered by limited viral gene expression during latency [3]. Because human gammaherpesviruses replicate poorly in cultured cells, our knowledge of this essential part of their lifecycle is limited. As with other herpesviruses, EBV replication begins in the nucleus where a set of highly conserved herpesvirus genes mediates genome replication and packaging into capsids. Egress from the nucleus is accomplished through primary envelopment at the nuclear membrane and subsequent de-envelopment in the cytoplasm. In the cytoplasm, capsids undergo secondary envelopment at the plasma membrane and simultaneously incorporate a proteinaceous layer beneath this envelope called the tegument. Although some of the genes responsible for EBV virion morphogenesis have been studied in detail, the role of most is inferred from studies of their homologs in alpha and beta herpesviruses [4]. Thus, gamma-specific genes, which have no homologs in the alpha and beta subfamilies, are the least well understood. In EBV, seven of the twelve gamma-specific genes, encode virion proteins: three glycoproteins (BMRF2, BDLF2, BDLF3 [gp150]) and four tegument proteins (BKRF4, BRRF2, BLRF2, BNRF1) [5], [6]. The BMRF2-BDLF2 glycoproteins have been shown to form a heterodimeric complex that facilitates direct cell to cell spread of EBV [7]. Whether the gamma-specific tegument proteins form similar complexes that adapt the virion morphogenesis program to the gammaherpesvirus niche is unclear. In an earlier study we identified a protein-protein interaction between the gamma-specific BLRF2 and BNRF1 tegument proteins [8] and sought to further characterize the composition of the BLRF2 complex.

The BLRF2 ORF encodes a 162 amino acid phosphoprotein with an apparent molecular weight of 23 kDa. Although not a capsid protein [6], it is a tightly capsid associated tegument protein and referred to in immunologic studies as viral capsid antigen (VCA)-p23. ELISA positivity for VCA-p23 is a sensitive and specific tool for diagnosing EBV infection [9]. Structural studies of ORF52, the BLRF2 homolog in murine herpesvirus 68 (MHV68), have revealed the protein to be comprised of a central dimerization domain from which the N and C terminal domains project, potentially to mediate protein-protein interactions [10]. ORF52 is essential for MHV68 morphogenesis, with the null mutant exhibiting a defect in virion egress from the infected cell [11]. The biochemical basis for BLRF2/ORF52’s role in virion assembly is unknown. Our prior identification of an interaction between BLRF2 and BNRF1, suggests that BLRF2 may recruit other gamma-specific proteins into the tegument. In an effort to better understand the basis for BLRF2’s role in virion assembly we sought to identify additional proteins that associate with BLRF2 and to characterize BLRF2 complexes from cells undergoing EBV replication.

To permit the purification of authentic BLRF2 replication complexes, we developed a system in which FLAG-HA-tagged BLRF2 could be stably expressed in a cell line capable of being synchronously induced for EBV replication (P3HR1-ZHT). Using this system we demonstrated that BLRF2 is relocalized from the nucleus to the cytoplasm during the course of EBV replication. We identified the Serine/Arginine-rich protein kinase 2 (SRPK2) as a component of BLRF2 complexes and mapped this interaction to the BLRF2 C-terminus. We show that an RS motif within the BLRF2 C-terminus is a substrate for SRPK2, affects BLRF2 subcellular location, and is required for viral replication in an MHV68 complementation assay. Furthermore, our approach is amenable to the characterization of other EBV replication complexes particularly as it does not require the construction of a recombinant EBV genome.

## Materials and Methods

### Cell Culture

293T is a human embryonic kidney cell line and HeLa is a cervical cancer cell line. P3HR1-ZHT FLAG-HA-GFP and FLAG-HA-BLRF2 cells were derived from P3HR1-ZHT cells [12] transfected with pMSCV-FLAG-HA-GFP or pMSCV-FLAG-HA-BLRF2, followed by continuous puromycin selection. All cell lines were cultured in Dulbecco’s modified Eagle’s (Gibco, Grand Island, NY) or RPMI 1640 (Gibco) medium supplemented with L-glutamine (Gibco), penicillin-streptomycin (Gibco) and 7.5% or 10% fetalplex (Gemini Bio-Products, West Sacramento, CA). FLAG-HA-BLRF2, FLAG-HA-GFP and P3HR1-ZHT wild-type cells were induced with 400 nM 4-Hydroxytamoxifen (4HT) (Sigma, St. Louis, MO) for 72 h and harvested for experiments and analysis.

### Plasmids

BLRF2 and BNRF1 entry clones, lacking stop codons to allow C-terminal fusions, have been described previously [8]. GFP entry clone was produced by amplifying the coding region from pEGFP-C1 (Clontech, Mountain View, CA) using the primers (ggggacaactttgtacaaaaaagttggcatggtgagcaagggcgaggagctg and ggggacaactttgtacaagaaagttggcttgtacagctcgtccatgccgag). BLRF2 (1–162) was amplified using the primers N1 (ggggacaactttgtacaaaaaagttggcatgtcagctccacgcaaagtcag) and C162stop (ggggacaactttgtacaagaaagttggtcaatcagaaatttgcactttctttgc). BLRF2 (1–130) was amplified using the reverse C130stop primer (ggggacaactttgtacaagaaagttggtcactcaccagggctgggttggcc) and BLRF2 (42–162) with forward N42 primer (ggggacaactttgtacaaaaaagttggcagagggggtgcctgtgcctcg), each paired with appropriate forward or reverse primers annealing in the vector backbone. The BLRF2 (SRS-ARA) point mutant was constructed by PCR mutagenesis using forward and reverse primers in the vector and internal primers (cgacgtgcccgcGcccgcGcccggggacgtgaagcaag and cacgtccccgggCgcgggCgcgggcacgtcgggtggc) containing the desired substitution (codon underlined, mutation in capital letters). Final PCR products were cloned into the pDONR223 gateway vector by BP recombinase reaction (Invitrogen, Carlsbad, CA) to generate Gateway compatible entry clones. Gateway entry clones for SRPK2, RTDR1, ZBED1, SNX-14b, PNRC2, MAGEA2B, DVL2, ZNF529, SRPRB, CASP1 and SNX-14a were provided by M. Vidal (Center for Cancer Systems Biology (CCSB), Dana Farber Cancer Institute, Boston, MA). Expression clones were subsequently generated using LR recombinase into the destination vectors pDEST27 (Invitrogen), Gateway converted pSG5-FLAG [13], pDEST-myc-eGFP [8] and/or MSCV-N-FLAG-HA-IRES-PURO [14] following the manufacturer’s instructions.

### Antibodies

The following antibodies were used for western blotting and immunofluorescence: Mouse monoclonal antibodies against GST (B14; Santa Cruz Biotechnology, Santa Cruz, CA), FLAG (M2; Sigma), alpha-Tubulin (B-5-1-2; Sigma), Phospho-SR (Ab104 collected from CRL-2067 hybridoma mouse cells; ATCC), EBV Rta (8C12; Argene, Varilhes, France), EBV Zta (AZ-69; Argene); rabbit polyclonal antibodies against EBV BNRF1 (a kind gift of Henri-Jacques Delecluse, German Cancer Research Centre, Heidelberg, Germany), EBV BLRF2 (SLO25-1, generous gift from George Miller, Yale University School of Medicine, New Haven, CT); goat polyclonal anti-GFP (T-19; Santa Cruz Biotechnology) and anti-lamin B (C-20; Santa Cruz).

### Western Blot Analysis

Protein samples were separated by sodium dodecyl sulfate (SDS)-polyacrylamide gel electrophoresis, blotted onto nitrocellulose membrane, and probed with appropriate antibodies. After extensive washing, membranes were incubated for 1 h with appropriate horseradish peroxidase conjugated secondary antibodies (Jackson Immuno Research, West Grove, PA) before being washed again, developed with chemiluminescence reagent (Perkin Elmer, Waltham, MA) and visualized on a KODAK Image Station 4000R (Kodak Molecular Imaging Systems, Rochester, NY) or Gel Logic 4000pro (Carestream Molecular Imaging, Rochester, NY).

### Yeast Two-hybrid

Protein-protein interactions were assessed using a bait/prey yeast mating strategy as described previously [15]. Briefly, BLRF2-AD transformed *MAT* **a** haploid yeast were mated with *MAT*α haploid yeast carrying DB-human hybrid proteins from the human ORFeome v 5.1 collection (∼15,000 full-length human ORFs; http://horfdb.dfci.harvard.edu/hv5/). Primary screens were completed twice and interacting clones were identified by growth on selective media. Interacting proteins were identified by sequencing and verified by retesting.

### GST Pull-down Assay

Transfected 293T cells were lysed in 1% NP-40 lysis buffer [50 mM Tris **pH** **7**.5, 150 mM NaCl, 1.5 mM EDTA, 1% NP-40, 3% glycerol, 10 mM NaF, 0.5 mM PMSF, 1% Aprotinin (Sigma), 2 mM Na_4_P_2_O_7_] and cleared by centrifugation at 16,000 g for 10 min. Supernatants were incubated with anti-Glutathione Sepharose 4B agarose (GE Healthcare Biosciences, Pittsburgh, PA) for 2 hours at 4°C. The agarose was washed extensively with 1% NP-40 lysis buffer and the precipitated proteins analyzed by western blotting.

### Cellular Fractionation

Cells were fractionated with a five-step subcellular protein fractionation kit (Thermo Scientific, Rockford, IL) according to the manufacturer’s recommendations. Alternatively cells were fractionated using either a one or two-step digitonin based protocol. Approximately 750,000 cells were lysed in 750 µl of Digitonin Extraction Buffer pH6.8 [0.015% Digitonin, 300 mM sucrose, 100 mM NaCl, 10 mM PIPES, 3 mM MgCl_2_, 1 mM PMSF, 3 mM EDTA, 10 mM 2-Mercaptoethanol] supplemented with one tablet of complete protease inhibitor cocktail (Roche, Mannheim, Germany) per 50 ml and 1 µl RNAse Out recombinant ribonuclease inhibitor (Invitrogen) per 10 ml and centrifuged at 480 g for 3 minutes and the soluble fraction was removed (Fraction-C). In the two step procedure, pellets were then lysed in Triton X-100 and centrifuged at 5,000 g for 20 minutes and the supernatant removed (Fraction-M1) and the pellet lysed again in Cell Lysis Buffer [50 mM Tris pH 7.5, 140 mM KCl, 10 mM NaF, 1.5 mM EDTA, 0.5% NP-40, 5% glycerol, supplemented with protease inhibitors as above], clarified by centrifugation twice at 11,000 g for 10 minutes and once at 16,000 g for 20 minutes at 4°C. Again the supernatant was removed (Fraction-S1) and the pellet resuspended in SDS loading buffer (Fraction-P1). In the one step procedure, the Digitonin pellets were lysed with Cell Lysis Buffer, the supernatant removed (Faction-S2), and the pellet resuspended in SDS loading buffer (Fraction-P2).

### Tandem Affinity Purification

Approximately 300 million cells were fractionated using the one-step Digitonin fractionation procedure described above to remove the cytosol and the remaining fraction incubated with washed anti-FLAG M2 agarose (Sigma) for 4 hours at 4°C. Following the incubation the beads were extensively washed in 0.5% NP-40 lysis buffer containing inhibitors and eluted with 0.8 mg/ml FLAG peptide (Sigma) in 0.5% NP-40 lysis buffer twice at 4°C for 45 minutes and once at 37°C for 45 minutes. The pooled FLAG elutions were then incubated overnight at 4°C with anti-HA agarose (Santa Cruz Biotechnology) followed by extensive washing and two elutions with 1.0 mg/ml HA peptide diluted in 0.05% NP-40 buffer for 45 minutes at 37°C. 10% of the pooled eluted proteins were separated by SDS-PAGE and analyzed by Silver Quest silver stain kit (Invitrogen) and the rest was analyzed by LC-MS/MS.

### LC-MS/MS

Purified protein complexes were denatured with 0.1% RapiGest (Waters, Milford, MA) and reduced with 10 mM DTT at 56°C for 30 minutes. Reduced cysteines were alkylated with 20 mM iodoacetamide at room temperature for 20 minutes in the dark. The samples were digested at 37°C overnight using 1 µg of trypsin. Following digestion, RapiGest was removed by acid cleavage and centrifugation according to manufacturer’s recommendations. Tryptic peptides were sequentially purified by batch-mode reverse-phase C18 and strong cation exchange SCX chromatography (using Poros 10R2 and Poros 20HS chromatography media respectively, Applied Biosystems, Carlsbad, CA). Purified peptide samples were loaded onto a pre-column (100 mm I.D.; packed with 4 cm POROS 10R2, Applied Biosystems) using a NanoAcquity Sample Manager and UPLC pump [16]. After loading, the peptides were gradient eluted (1–30% B in 45 minutes; buffer A: 0.2 M acetic acid, buffer B: 0.2 M acetic acid in acetonitrile) at a flow rate of ∼50 nl/minute to an analytical column (30 mm I.D. packed with 12 cm Monitor 5 mm C18 from Column Engineering, Ontario, CA), and introduced into an LTQ-Orbitrap XL mass spectrometer (ThermoFisher Scientific, Waltham, MA) by electrospray ionization (spray voltage = 2200 V). The mass spectrometer was programmed to operate in data dependent mode, such that the top 8 most abundant precursors in each MS scan (detected in the Orbitrap mass analyzer, resolution = 60,000) were subjected to MS/MS (CAD, linear ion trap detection, collision energy = 35%, precursor isolation width = 2.8 Da, threshold = 20,000). Dynamic exclusion was enabled with a repeat count of 1 and a repeat duration of 30 sec. Orbitrap raw data files were processed within the multiplierz software environment [17]. MS spectra were recalibrated using the background ion (Si(CH_3_)_2_O)_6_ at m/z 445.12+/−0.03 and converted into a generic Mascot file format (.mgf). Spectra were searched using Mascot version 2.3 against 4 appended databases of: i) human protein sequences (downloaded from RefSeq on July 11 2011); ii) a database of EBV proteins; iii) a database of common lab contaminants and iv) a decoy database generated by reversing the sequences from the human database. Search parameters specified a precursor ion mass tolerance of 1 Da, a product ion mass tolerance of 0.6 Da, oxidation of methionine (M, +16 Da) and cysteine alkylation (C, +57 Da). The lists of peptide hits from the Mascot searches were filtered to exclude precursors with a mass error greater than 5 ppm. Sequence matches to the decoy database were used to enable a 1% false discovery rate (FDR) filtering of the resulting peptide identifications.

### Pathway Enrichment Analysis

Enrichment of KEGG pathways was calculated for all proteins identified by TAP-MS using DAVID (http://david.abcc.ncifcrf.gov/) [18].

### Immunofluorescence Analysis

Suspension cells were harvested at 200 g for 3 minutes, stained with Draq5 (Biostatus Limited, Shepshed, UK) at 37°C for 15 minutes and streaked onto coverslips. Adherent cells were seeded and transfected on Poly-D-lysine (BD Biosciences, San Jose, CA) coated coverslips. All cells were fixed with 1% (w/v) paraformaldehyde in PBS and permeabilized with 0.1% (v/v) TritonX-100 in PBS containing 1 mM glycine. Cells were blocked with 5% bovine serum in PBS and incubated with primary antibodies diluted in PBS plus 1% (w/v) bovine serum albumin (BSA) according to the manufacturers’ instructions. Secondary antibodies, mouse and rabbit Alexa Fluor 488 (Molecular Probes, Eugene, OR) diluted in PBS plus 1% (w/v) BSA. The slides were mounted with ProLong Gold antifade reagent (Molecular Probes) and imaged with a laser scanning Zeiss Axioskop PCM 2000 (Zeiss, Oberkochen, Germany).

### MHV68 Complementation Assay

Complementation of the replication defective MHV68 52S mutant was performed as previously described [19]. Briefly, MHV-68 52S BAC DNA (1500 ng/well) plus empty vector (500 ng/well) or plasmid DNA (500 ng/well) expressing BLRF2 or BLRF2-ARA mutant was transfected into subconfluent 293T cells in 12 well plates with PEI solution, in serum free DMEM medium (Gibco) without antibiotics with medium changed after 12 hours. Four days after transfection, supernatant was collected and cleared of any debris by centrifugation at 1500 *g*. Released viral DNA was quantified by qPCR. Using primers that amplify a 67 bp fragment of the MHV-68 ORF65 coding region (GTCAGGGCCCAGTCCGTA and TGGCCCTCTACCTTCTGTTGA).

## Results

### Identification of BLRF2 Interacting Proteins by Yeast Two-hybrid Assay

Our earlier interactome study found that BLRF2 interacted with the EBV tegument protein BNRF1, but did not identify any BLRF2 interacting cell proteins [8]. However, that yeast two-hybrid assay only screened a limited number of transformants (∼10^6^) from a cDNA library. To more comprehensively identify BLRF2 interacting proteins, we searched the human ORFeome v5.1 collection consisting of ∼15,000 full length human ORFs [20], [21] using BLRF2 in both the bait and prey configuration. Two cell proteins (DVL2 and ZBED1) were found to interact with gal4-DB-BLRF2 and were recently published as part of a larger dataset [22]. Eleven (MAGEA2B, SRPK2, SRPRB, ZNF529, CASP1, CCL3L3, NLE1, PNRC2, RTDR1, SLC6A13 and SNX14) proteins interacting with gal4-AD-BLRF2 are reported here (Table 1).

**Table 1:** BLRF2 interacting proteins.

	Y2H	GST-BLRF2			
Prey	P/B	Tested	1–162	1–130	42–162
BNRF1	P	+	+	−	+
DVL2	B	+	+	−	+
ZBED1	B	+	+	−	+
SRPK2	P	+	+	−	+
SRPRB	P	+	+	−	+
MAGEA2B	P	+	−		
CASP1	P	+	−		
ZNF529	P	−			
SSL3L3	P	−			
NLE1	P	−			
PNRC2	P	−			
RTDR1	P	−			
SLC6A13	P	−			
SNX14	P	−			

P Prey; B Bait.

+ Positive;

− Negative.

**Figure 1:**
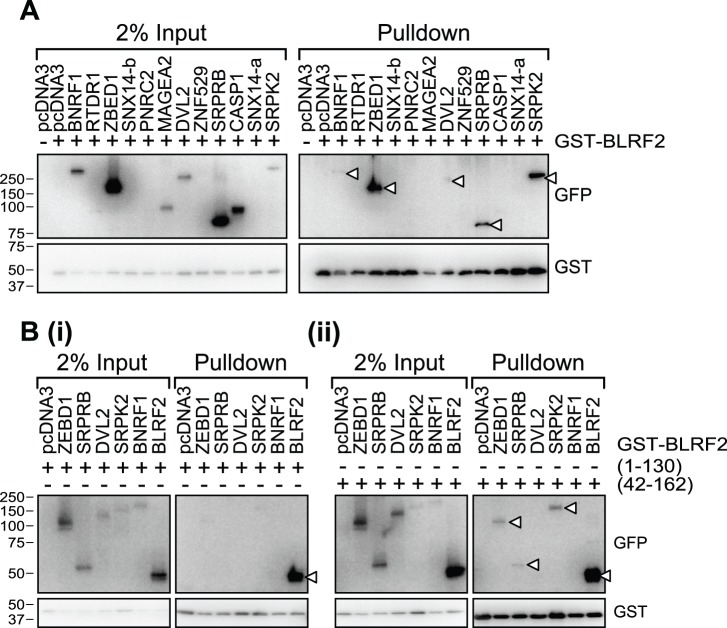
BLRF2 C-terminus mediates interaction with host and EBV proteins. (A) GST pull-down assay to confirm binding of putative BLRF2 interacting proteins identified in a yeast two-hybrid assay. Lysates from 293T cells transfected with GST-BLRF2 and the indicated GFP tagged proteins were captured with GST-agarose, washed, resolved by SDS page and proteins detected by western blotting with anti-GFP (upper panels) and anti-GST antibodies (lower panels). Input lysates (2%) are shown in the left panels. (B) Mapping of BLRF2 binding partners confirmed in (A) to the BLRF2 N-terminus (GST-BLRF2 aa1–130 (i)) or C-terminus (GST-BLRF2 aa42–162 (ii)).

To test these potential interactions in mammalian cells, co-affinity precipitation experiments were performed. We obtained full-length entry clones for eleven of the putative BLRF2 interacting proteins, identified in this or prior screens, seven of which could be expressed as GFP fusions in human embryonic kidney 293T cells. GST-tagged BLRF2 was co-expressed in 293T cells with each of the putative interacting proteins and five of them, BNRF1, ZBED1, DVL2, SRPRB and SRPK2, co-precipitated with gst-BLRF2 (Fig. 1A and Table 1).

In order to map the interacting domain within BLRF2, overlapping N (aa 1–130) and C (aa 42–162) terminal fragments of BLRF2 were generated and tested by co-expression in 293T cells and GST co-precipitation assays for the five interacting proteins. ORF52 is known to homo-dimerize via its central domain corresponding to BLRF2 aa 66–122 [10], therefore as a control, GFP-BLRF2 was co-expressed and tested for self-association with both the N and C-terminal fragments. The N-terminal fragment (aa 1–130) precipitated GFP-BLRF2 but none of the other proteins were found to interact robustly with the BLRF2 N-terminus (Fig. 1Bi). The C-terminal fragment (aa 42–162) co-precipitated GFP-BLRF2 and also interacted with ZBED1, SRPRB and SRPK2 (Fig. 1Bii and Table 1). Thus, the BLRF2 C-terminus can mediate interactions with a number of cell proteins.

### Characterization of BLRF2 during EBV Replication

**Figure 2:**
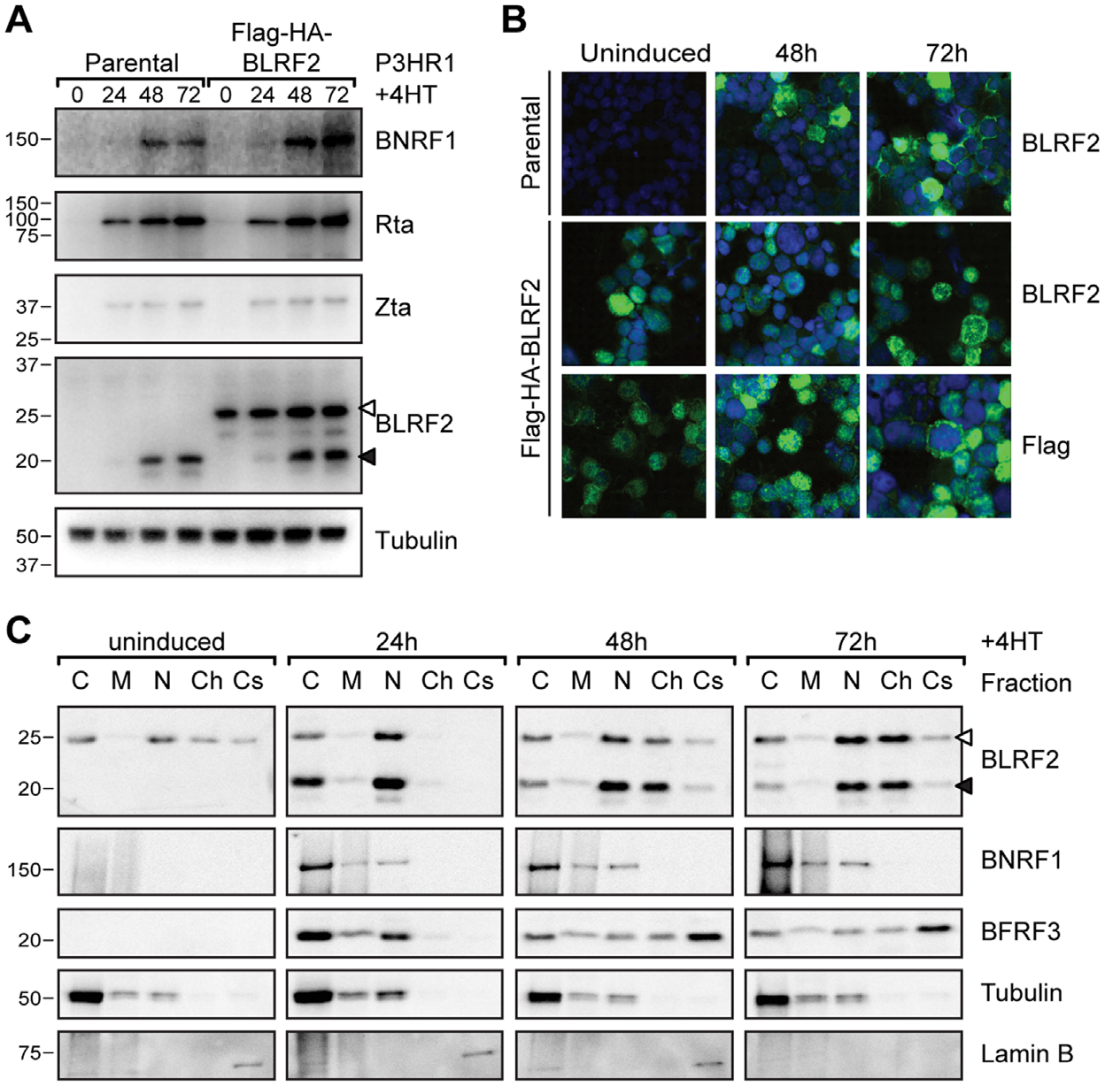
Characterization of BLRF2 during EBV replication. (A) Time course of EBV protein expression using whole cell lysates from P3HR1-ZHT cells (parental) or P3HR1-ZHT cells stably expressing FLAG-HA-BLRF2 induced with 4HT for 0, 24, 48 or 72 hours. Detection of tubulin serves as loading control. Endogenous BLRF2 is indicated with a solid arrowhead and FLAG-HA-BLRF2 with an open arrowhead. (B) Immunofluorescence microscopy to determine BLRF2 localization during EBV replication in P3HR1-ZHT cells (parental, top panel) or P3HR1-ZHT cells stably expressing FLAG-HA-BLRF2 (FLAG-HA-BLRF2, bottom panels), either uninduced (left) or induced with 4-Hydroxytamoxifen (4HT) for 48 hours (middle) or 72 hours (right). Anti-BLRF2 and anti-FLAG staining are shown in green and DNA staining is shown in blue. (C) Subcellular fractionation of EBV proteins and control cell proteins from P3HR1-ZHT cells stably expressing FLAG-HA-BLRF2 induced for replication with 4HT for 0, 24, 48 or 72 hours. Equal relative amounts of the cytosol (C), membrane and organelles (M), nucleus (N), chromatin bound (Ch) and cytoskeletal (Cs) fractions were probed for the indicated proteins. Tubulin served as a control for the cytosol fraction and Lamin B for the cytoskeleton fraction. As for panel A, endogenous and FLAG-HA tagged BLRF2 are indicated with filled and open arrowheads, respectively.

To characterize endogenous BLRF2 complexes during EBV replication, FLAG-HA-BLRF2 was stably expressed in EBV positive P3HR1-ZHT Burkitt Lymphoma cells [12]. These cells express Zta fused to the 4-hydroxy-tamoxifen (4HT)-dependent mutant estrogen receptor hormone binding domain (ZHT) [6] and can be synchronously induced for EBV replication by the addition of 4HT ligand. To ensure that the constitutive expression of BLRF2 did not affect the ability of the P3HR1 EBV episomes to replicate, cells were induced with 4HT for 0, 24, 48 or 72 hours and whole cell lysates blotted for EBV protein expression. As expected, the immediate early gene products Rta and Zta were detectable within 24 hours of 4HT addition (Fig. 2A). BLRF2 and BNRF1 were expressed after 24 hours of induction and increased over the time course. Notably, in these cells FLAG-HA-BLRF2 was expressed at levels comparable to endogenous BLRF2.

To further investigate whether tagged BLRF2 mirrored the endogenous protein, BLRF2 localization was observed over time by immunofluorescence (IF) microscopy. Endogenous BLRF2 appears predominantly nuclear when first expressed at 24 hours (not shown) and 48 hours post induction of P3HR1-ZHT cells (Fig. 2B) and began to accumulate in the cytoplasm as replication progressed (72 hours). Constitutively expressed tagged BLRF2 demonstrated a similar pattern, localizing primarily to the nucleus in the absence of EBV replication and redistributing to the cytoplasm following the induction of replication.

Potential differences between tagged and endogenous BLRF2 were further investigated by performing subcellular fractionation on FLAG-HA-BLRF2 P3HR1-ZHT cells induced for EBV replication for 0, 24, 48 or 72 hours. Tagged BLRF2 was found predominately in the nuclear and cytoplasmic fractions before induction and at 24 hours post induction. During replication, BLRF2 accumulated in the chromatin fraction and small amounts were also observed in the cytoskeletal fraction. The BNRF1 tegument protein was detected 24 hours post induction, but remained mostly in the cytosol with smaller amounts fractionating with the membrane/organelle and nucleoplasmic fractions. BFRF3, a capsid protein, was also initially found in the cytosol and nuclear fractions at 24 hours post induction, but by 72 hours post induction was found predominantly in the insoluble (cytoskeletal) fraction (Fig. 2C). The appearance of the BLRF2, BNRF1, and BFRF3 virion proteins in distinct fractions argues that no single fraction corresponds to intracellular virions. Rather, the extraction procedure was simultaneously dissembling nacient virions and cellular compartments. Since it is highly improbable that BLRF2, a known virion component, does not leave the nucleus, we suspect that virion associated BLRF2 may co-fractionate with nuclear BLRF2 in this assay. In any event, observed changes in localization throughout replication are the same for FLAG-HA-BLRF2 and endogenous BLRF2.

### Identification of BLRF2 Protein Complexes in Cells Undergoing EBV Replication

**Figure 3:**
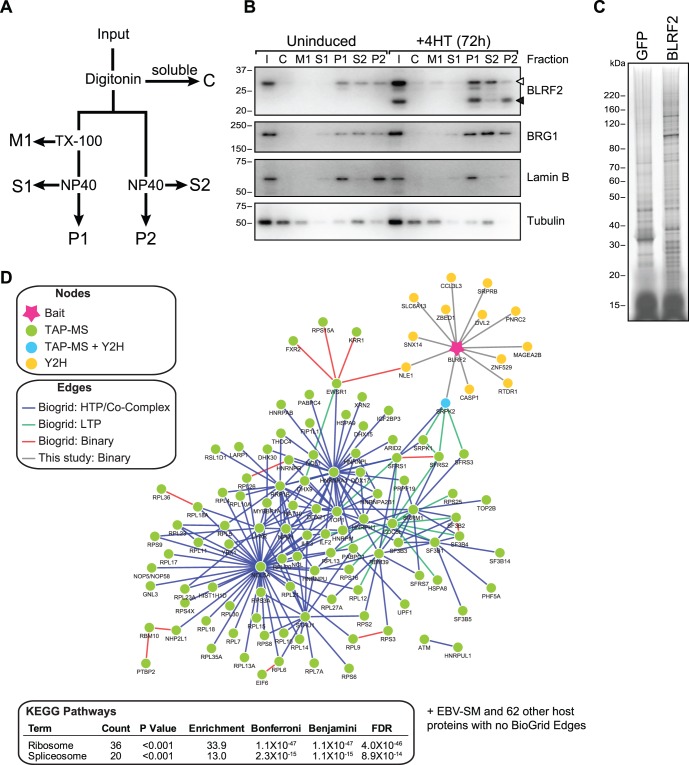
BLRF2 forms protein complex with SRPK2 and host RNA splice factors. (A) Schematic of two fractionation procedures tested to extract BLRF2 complexes. The cytosol (C) was removed by Digitonin extraction and split between two procedures. Procedure 1 was a three step process in which the membrane and organelles (M1) were collected after Triton X-100 (TX-100) lysis, the soluble fraction after NP40 lysis (S1) and the remaining insoluble pellet (P1). Procedure 2 was only two steps in which the soluble fraction (S2) was collected after NP40 lysis and the remaining insoluble pellet (P2). (B) Western blot analysis of the fractions obtained using procedure described in (A). BLRF2 extraction was monitored using rabbit polyclonal anti-BLRF2 antibody. Endogenous BLRF2 is indicted with a filled arrowhead and FLAG-HA-BLRF2 with an open arrowhead. Fraction composition was also assessed by western blotting for control cell proteins BRG1 (nuclear and chromatin bound), lamin B (cytoskeleton) and tubulin (cytoplasmic). (C) Silver stain gel of 10% of the final elutions from a tandem affinity purification of FLAG-HA-GFP and FLAG-HA-BLRF2 stable P3HR1-ZHT cell lines. Molecular weights of size markers are shown (left). (D) Network representation of interacting proteins identified in TAP-MS and Y2H generated by Pathway Palette and the BioGrid database. EBV proteins are shown as stars and host proteins as circles. The bait (BLRF2) is shown in pink. Interacting proteins are colored based on the technology that identified them (TAP – green; Y2H – yellow; Both – blue). Edges are colored based on the type of evidence used to infer the interaction (Co-complex - blue edges; Binary – red). EBV protein interactions are all binary as described in the text and host-host interaction data is derived from the Biogrid database. Only the connected components are shown. The table shows enrichment of KEGG Pathways for proteins identified by TAP-MS.

To detect BLRF2 interactions that occur in cells during EBV replication we chose to purify BLRF2 complexes from cells at 72 hours post induction. Because preliminary attempts (not shown) to purify BLRF2 were limited by contamination with highly abundant cytoplasmic proteins, we removed the cytosol using digitonin, and then tested two different extraction procedures (Fig. 3A). For the first procedure, the membrane and organelles were removed by an ice-cold Triton X-100 (TX-100) extraction and the pellet subsequently lysed in a NP40 buffer. The second procedure is similar but lacks the TX-100 extraction step. Surprisingly, although little to no BLRF2 was extracted by TX-100 (Fig 3B, M1), the addition of this step resulted in almost all of the BLRF2 being lost in the insoluble pellet (Fig 3B, compare P1 and S1). The second procedure solubilized approximately 50 percent of the FLAG-HA-BLRF2 and about 10 percent of endogenous BLRF2 (Fig. 3B, compare P2 and S2). Therefore, BLRF2 associated protein complexes were purified from the S2 fraction in subsequent biochemical analysis.

Tandem Affinity Purification (TAP), first by FLAG IP and FLAG peptide elution followed by HA IP and elution, was performed on S2 lysates from FLAG-HA-BLRF2 and FLAG-HA-GFP P3HR1-ZHT cells that had been induced with 4HT for 72 hours. Ten percent of the final elutions were separated by SDS-PAGE and visualized by silver stain and indicate that BLRF2 precipitated specific protein complexes compared to the GFP background control (Fig. 3C). To identify the interacting proteins, the remainder of the purified complexes were analyzed by LC-MS/MS and a total of 166 putative interacting proteins were unambiguously identified. The final dataset of protein associations identified by the TAP, along with the previously identified Y2H interactions were analyzed using Pathway Palette [23]. Published protein interactions were retrieved from the BioGrid database [24] and used to highlight putative complexes among proteins associated with BLRF2 (Fig. 3D). To identify pathways that are targeted by BLRF2 we investigated the enrichment of KEGG pathways among the BLRF2 interacting proteins. BLRF2 showed a significant enrichment for proteins annotated in the Ribosome (36 proteins, P<0.001) and the Splicesome (20 proteins, P<0.001). Despite the fundamental differences between Y2H and TAP-MS, and the limitations in both assays’ sensitivity, one interacting protein, SRPK2 was found by both screening approaches and had been confirmed by the GST pull-down assays (Fig. 1), therefore we decided to explore this interaction further.

### BLRF2 is a Substrate for SRPK2

**Figure 4:**
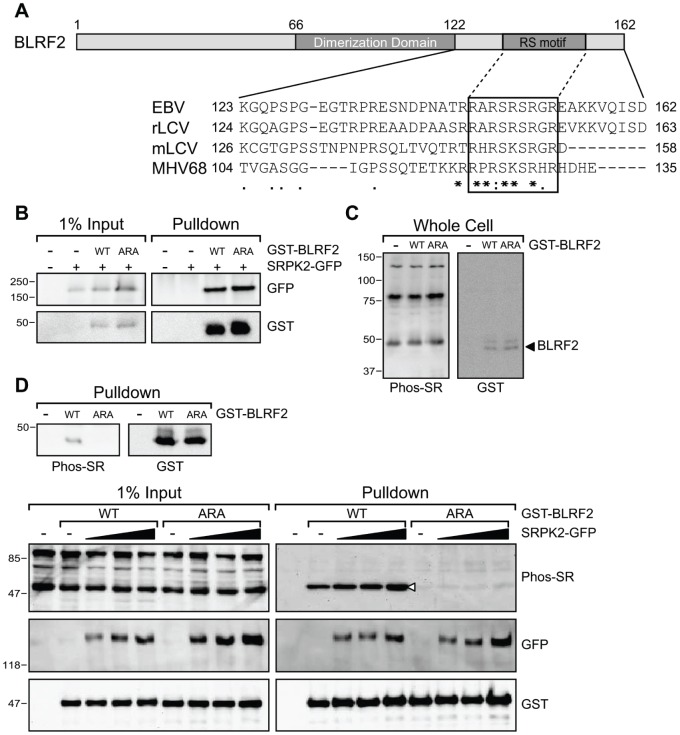
SRPK2 phosphorylates BLRF2. (A) Schematic of BLRF2 indicating the central dimerization domain (aa 66–122) and the C-terminal RS motif. ClustalW alignment of the C-termini of BLRF2 (aa 123 to 162) and its homologs from the rhesus and marmoset lymphocryptoviruses and murine gammaherpesvirus 68. Degree of conservation is shown at the bottom (* - identical, **:** - high,**.** - moderate). A conserved basic domain containing the putative RS motif is highlighted by the box. (B) GST pull-down of 293T cells transfected with GST-BLRF2 wild-type (WT) or GST-BLRF2 SRS-ARA mutant (ARA) in the presence or absence of GFP-SRPK2. Western blot analysis using anti-GFP (top panels) and anti-GST (lower panels) antibodies. Input lysates (1%) are shown in the left panels. (C) Western blots of transfected 293T whole cell lysates probed with anti-Phospho-SR antibody (left) and anti-GST antibody (right). (D) Western blots of GST pull-downs from 293T cells transfected with GST-BLRF2 wild-type or ARA mutant. Phosphorylated GST-BLRF2 is shown in the left panel with anti-Phospho-SR antibody and total GST-BLRF2 level is shown by anti-GST antibody (right panel). (E) Western blot of GST pull-downs of 293T cells transfected with GST-BLRF2 wild-type and ARA mutant along with increasing amounts of SRPK2-GFP. Phosphorylated protein is detected by anti-Phosho-SR antibody (top panels), anti-GFP antibody showed SRPK2-GFP (middle panels) and anti-GST antibody showed total BLRF2 (bottom panels). Input lysates (1%) are shown in the left panels.

SRPK2 is a member of the serine/arginine-rich protein kinase family that phosphorylates arginine/serine (RS) rich motifs found predominantly in non-snRNP splicing factors called “SR proteins.” [25]. In addition to a role in regulating pre-mRNA splicing by phosphorylating SR proteins, SRPKs also play an important role in cell proliferation and apoptosis [26], [27]. SRPK2 has been shown to induce apoptosis via an upregulation of nuclear cyclin D1 and to trigger cell cycle progression in post-mitotic neurons [28]. SRPK2 bound to the C-terminus (aa 42–162) but not the N-terminus (aa 1–130) of BLRF2, suggesting that the residues after the dimerization domain may be important in regulating the interaction. ClustalW [29] alignment of BLRF2 with its homologs from the closely related rhesus and marmoset lymphocryptoviruses and ORF52 from the more distantly related murine gammaherpesvirus 68 [30], [31], [32] revealed a conserved C-terminal basic domain. In BLRF2, this domain contained two arginine-serine (RS) repeats that could be SRPK2 substrates. (Fig. 4A). In order to investigate the significance of these conserved RS motifs in BLRF2, serine 148 and 150 were mutated to alanines (ARA mutant). GST pull-down experiments with BLRF2 wild-type and ARA point mutant, revealed that the interaction with SRPK2 was not affected by the mutation (Fig. 4B).

To investigate whether the BLRF2 RS motif is a target for SRPK2 phosphorylation, wild-type or ARA mutant BLRF2 was expressed in 293T cells and whole cell lysates were then probed with a phospho-SR specific antibody. Because of the large number of SR splicing factors recognized by this antibody, it was difficult to unambiguously establish that the BLRF2 RS motif was phosphorylated by probing whole cell lysates (Fig. 4C). To circumvent this and enrich for BLRF2, GST pull-downs were performed on lysates from cells expressing GST-BLRF2 wild-type or GST-BLRF2 ARA mutant and probed with the same antibody. A GST blot shows strong expression and pull-down of both wild-type and ARA mutant, but only the wild-type protein was detected by the anti-phosphoSR antibody (Fig. 4D). Because the ARA mutant still interacts with SRPK2, this data suggests that BLRF2 serines 148 and/or 150 are phosphorylated by SRPK2.

To further confirm the role of SRPK2 in BLRF2 phosphorylation, increasing amounts of SRPK2-GFP were expressed in 293T cells co-transfected with BLRF2 wild-type or ARA mutant. Total BLRF2 protein levels did not change but phospho-SR BLRF2 levels increased by as much as two-fold with SRPK2-GFP co-expression (Fig. 4E).

### A BLRF2 Mutant Lacking SRPK2 Phosphorylation Sites cannot Complement ORF52 Null MHV68 Replication

**Figure 5:**
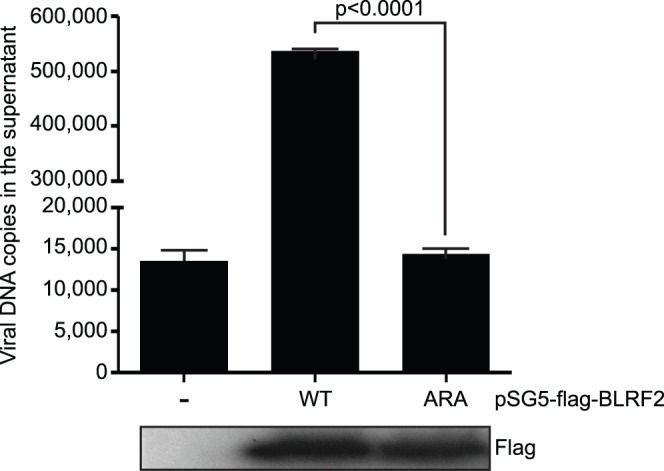
BLRF2 rescues ORF52 null MHV68 replication, but the BLRF2-ARA mutant cannot. (A) Complementation assay measuring MHV68 virion release by quantitative PCR into supernatants of 293T cells co-transfected with replication defective MVH68 ORF52 null BAC and empty vector, wild-type FLAG-BLRF2, or FLAG-BLRF2-ARA mutant. Viral DNA was quantified four days post-transfection and the results shown are representative of two independent experiments performed in triplicate. Western blot with anti-flag antibody shows BLRF2-WT and –ARA expression levels.

An MHV68 ORF52 null mutant is defective in virion egress but this defect can be overcome by complementation with wild-type ORF52 [11] The function of BLRF2 and its ORF52 homologs in other gammaherpesviruses is sufficiently conserved that EBV BLRF2 can also complement the replication defect of an ORF52 null MHV68 genome [19]. We exploited this observation to test the relevance of the RS motif for BLRF2 function. In the absence of ORF52 expression, MHV68 DNA replication and encapsidation is normal, but virions accumulate in the cytoplasm and release of extracellular virions is extremely impaired [11]. When ORF52 null MHV68 is co-transfected with BLRF2 expression plasmid, virion release increased almost 40-fold (P<0.0001) as determined by measurement of viral DNA copies in the supernatant (Fig. 5). However, when the BLRF2 ARA mutant was cotransfected, MHV68 DNA was at nearly the same level as that seen with vector control. This is consistent with the RS motif, and likely its phosphorylation, playing an important role in BLRF2 function.

### Mutation of the BLRF2 SR Motif Decreases BLRF2 Nuclear Localization

**Figure 6:**
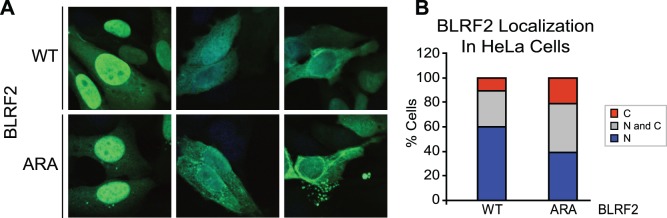
The BLRF2 ARA mutant exhibits increased cytoplasmic localization compared to wild-type BLRF2. (A) Immunofluorescence microcopy showing BLRF2 localization in HeLa cells transfected with BLRF2 wild-type or ARA mutant. Cells were stained with anti-BLRF2 antibody (green) and Draq5 DNA staining (blue). Examples of predominantly nuclear (left panels), mixed nuclear and cytoplamsic (middle panels), and predominantly cytoplasmic staining (right panels) are shown. (B) Summary of immunofluorescence analysis described in (A). The percentage of cells observed with predominantly nuclear (N) (blue), cytoplasmic (C) (red) or mixed (N and C) (gray) BLRF2 staining are shown.

Because SRPK2 phosphorylation of SR proteins enhances their entry into the nucleus [33], we investigated the possibility that SRPK2 regulates BLRF2 subcellular localization. Initially, pulldown experiments were preformed on cytoplasmic and nuclear fractions from FLAG-HA-BLRF2 P3HR1-ZHT cells induced for replication. SRPK2 was observed to efficiently co-precipitate with BLRF2 in both fractions (Fig. S1). Next, we examined the effect of the ARA mutation on BLRF2 subcellular distribution in transfected HeLa cells. At least 100 cells were observed and classified as showing exclusively nuclear, exclusively cytoplasmic, or mixed nuclear and cytoplamic BLRF2 staining. As observed before, wild-type BLRF2 was predominantly nuclear (60% of cells) or mixed nuclear and cytoplasmic (29%) with only 11% of cells having exclusively cytoplasmic staining (Fig. 6A & 6B). In contrast, the ARA mutant was exclusively nuclear in 39%, mixed nuclear and cytoplasmic in 40%, and exclusively cytoplasmic in 21% of cells. Thus, the ARA mutation resulted in a modest, but statistically significant shift of BLRF2 from the nucleus to the cytoplasm (chi-squared 9.3, P<0.009). Thus, SRPK2 mediated phosphorylation of BLRF2 has a modest effect BLRF2 nuclear localization that is unlikely to explain the dramatic effect of the ARA mutation on viral replication.

## Discussion

EBV replication and virion morphogenesis involves the coordinated action of highly conserved replication gene products with those found only in gammaherpesviruses. The roles played by these genes are only beginning to be explored, but studies of the murine gammaherpesvirus MHV68 suggest they are essential. Tegument proteins in particular appear to play a critical role as disruption of any one of the four (the homologs of EBV BNRF1, BLRF2, BKRF4, and BRRF2) abrogates replication [32]. By contrast, homologs of genes that encode EBV glycoproteins (BDLF2, BDLF3, BMRF2) were all dispensable. We have previously searched for binary protein-protein interactions among all the EBV proteins using a yeast two-hybrid assay [8]. In this report, we describe a system in which EBV replication protein complexes can be studied during EBV replication. This system offers two clear advantages over the alternative of generating recombinant EBV genomes. First, it is less labor intensive and hence, more amenable to scaling up to a high throughput analysis. Second, use of the P3HR1-ZHT cell line as a background, allows efficient and synchronous induction of EBV replication. Using the BLRF2 tegument protein as a prototype, we demonstrated that the epitope tagged protein could be expressed at levels comparable to that seen in EBV replication. In the course of EBV replication, FLAG-HA-BLRF2 moved from the nucleus to the cytoplasm in a manner indistinguishable from endogenous BLRF2. However, the solubility of the BLRF2 complexes proved a major obstacle to their characterization. Even with optimized extraction procedures, we were only able to solubilize about 50% of the FLAG-HA-BLRF2. Our inability to detect BNRF1 or other virion components in the purified complexes suggests that capsid associated BLRF2 complexes were either not extracted, disrupted, or both. Nevertheless, characterization of solubilized BLRF2 complexes revealed that SRPK2, which had been identified as a binary interacting partner of BLRF2 by yeast two-hybrid assay, interacted with BLRF2 during EBV replication in B lymphocytes.

SR proteins are highly conserved splicing factors comprised of N-terminal RNA-binding domains and arginine-serine rich C-termini (RS motifs) which are substrates for the SR protein kinases SRPK1 and SRPK2. RS motif phosphorylation by SRPKs has been shown to regulate SR protein subcellular localization, protein-protein interactions, protein-RNA interactions, and splicing catalysis [26], [27]. Our data demonstrates that the RS motif in the C-terminus of BLRF2 is a substrate for SRPK2 and that mutation of this motif alters BLRF2 nuclear/cytoplasmic partitioning. Further, mutation of this RS motif abrogates the ability of BLRF2 to complement the inactivation of its homolog (ORF52) in an MHV68 replication assay. SRPKs appear to be frequently targeted by replicating viruses [34], [35], [36], [37]. During herpes simplex virus (HSV) replication, SR protein phophorylation is decreased and splicing inhibited, potentially due to relocalization of SRPK1 to the nucleus by the HSV ICP27 protein [37]. The EBV ICP27 homolog, SM, has been shown to interact with the SR protein SRp20 to direct specific alternative splice-site selection [38]. Viral targeting of SRPK also has effects that are independent of splicing. For example, SRPK1 and SRPK2 phosphorylation of the hepatitis B core protein is required for its stable association with viral genomic RNA [34]. Although we cannot exclude a role for BLRF2 in regulation of splicing, this seems an unlikely role for a gene expressed late during viral replication. The ability of RS motif phosphorylation to alter protein-protein interactions could play an important role in regulating BLRF2’s association with capsids or other tegument proteins. This reversible modification would allow regulation of BLRF2 protein binding to promote assembly of virions in a productively infected cell and facilitate virion disassembly during initial infection.

Although the precise function of BLRF2/ORF52 in gammaherpesvirus replication remains to be defined, current evidence suggests it plays a role in tegument acquisition and organization. The strong association of BLRF2 with EBV capsids may allow it to serve as an anchor for recruitment of other tegument proteins. This view is supported by ultrastructural studies during the abortive replication of MHV68 ORF52 null cells, in which the electron-dense proteinaceous structures normally found at the capsid-vesicle interface were absent during secondary envelopment [19]. When isolated, these immature viral particles lacked specific viral proteins, such as the gammaspecific tegument protein encoded by ORF45. Interactome studies of EBV, KSHV, and MHV68 have identified a total of thirteen viral proteins that interact with ORF52/BLRF2 in binary assays (Table 2) [8], [39], [40], [41]. Interestingly, interactions with divergent proteins (e.g., those encoded by gammaspecific genes) have been more consistently detected across multiple gammaherpesviruses than ORF52/BLRF2 interactions with conserved proteins. This may indicate a role for ORF52/BLRF2 in tethering gammaspecific tegument proteins to the capsid. Such tethering could fulfill at least two potential functions. First, it would bridge interactions with cellular machinery and the viral capsid as has been demonstrated for KSHV ORF45 which promotes KSHV egress by interacting with the kinesin motor protein KIF3A [42]. Second, ORF52/BLRF2 tethering could ensure delivery of tegument proteins to newly infected cells. For example, delivery of the BNRF1 has been shown to promote the establishment of EBV infection by binding to Daxx, displacing ATRX and disrupting PML nuclear bodies [43]. It will be important to determine whether and how each of these protein-protein interactions depends on phosphorylation of the BLRF2 RS motif. Inhibition of SRPK1 and SRPK2 has been explored as a potential therapy for HIV infection with limited success; however, these compounds may also prove useful for further exploring the BLRF2’s role in EBV replication [35].

**Table 2:** Gammaherpesvirus proteins reported to interact with ORF52/BLRF2.

	EBV	KSHV	MHV68	Conservation
ORF6/BALF2		+		α, β, γ
ORF26/BDLF1		+		α, β, γ
ORF31/BDLF4		+		β, γ
ORF33/BGLF2		+		α, β, γ
ORF34/BGLF3		+	+	β, γ
ORF39/BBRF3		+		α, β, γ
ORF45/BKRF4		+	+	γ
ORF47/BKRF2*		+		α, β, γ
ORF49/BRRF1		+		γ
ORF52/BLRF2	+	+	+	γ
ORF53/BLRF1		+		α, β, γ
ORF57/SM		+		α, β, γ
ORF59/BMRF1		+		α, β, γ
ORF60/BaRF1		+		α, β
ORF64/BPLF1*		+		α, β, γ
ORF69/BFLF2		+		α, β, γ
ORF75/BNRF1	+	+		γ

* Interaction with detected with ORF fragment.

## Supporting Information

Figure S1 **BLRF2 and SRPK2 associate in both the nucleus and cytoplasmic fractions of cells during EBV replication.** P3HR1 ZHT cells stably expressing flag-HA-BLRF2 were induced for replication by addition of 4-hydroxytamoxifen. After 48 hours, cells were harvested and either directly lysed in IP lysis buffer (T) or fractionated by hypotonic lysis followed by centrifugation into cytoplasmic (C) and nuclear fractions (N). Each fraction was immunoprecipitated for BLRF2 using flag beads (M2, Sigma) and after extensive washing, resolved by SDS page and blotted for BLRF2 (HA antibody) and SRPK2. Input lysates (2%) are shown for comparison and were probed for alpha tubulin and histone H2B to assess fraction purity.
(PPT)
