# Supplementary figures and images for: An RS Motif within the Epstein-Barr Virus BLRF2 Tegument Protein Is Phosphorylated by SRPK2 and Is Important for Viral Replication
Source: PLoS One. 2013 Jan 9;8(1):e53512. doi: 10.1371/journal.pone.0053512 (PMC3541133; doi:10.1371/journal.pone.0053512)

## Slide 1
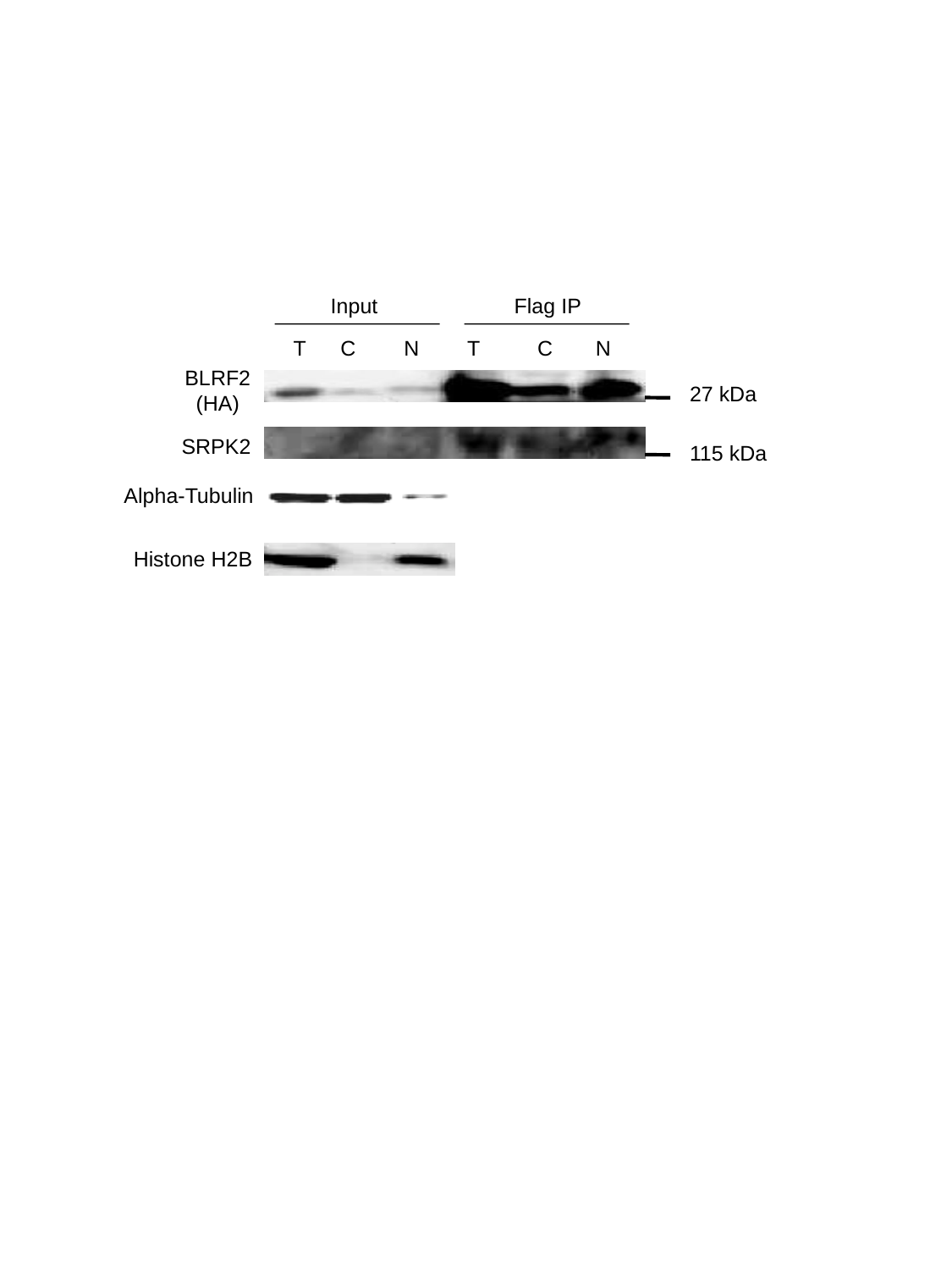

Input
Flag IP
T
C
N
T
C
N
BLRF2
(HA)
27 kDa
SRPK2
115 kDa
Alpha-Tubulin
Histone H2B

Supplement: Figure S1 — BLRF2 and SRPK2 associate in both the nucleus and cytoplasmic fractions of cells during EBV replication. P3HR1 ZHT cells stably expressing flag-HA-BLRF2 were induced for replication by addition of 4-hydroxytamoxifen. After 48 hours, cells were harvested and either directly lysed in IP lysis buffer (T) or fractionated by hypotonic lysis followed by centrifugation into cytoplasmic (C) and nuclear fractions (N). Each fraction was immunoprecipitated for BLRF2 using flag beads (M2, Sigma) and after extensive washing, resolved by SDS page and blotted for BLRF2 (HA antibody) and SRPK2. Input lysates (2%) are shown for comparison and were probed for alpha tubulin and histone H2B to assess fraction purity. (PPT) [file pone.0053512.s001.ppt]
